# Integrative traditional Chinese medicine for lumbar disc herniation after surgery: A protocol for systematic review and meta-analysis

**DOI:** 10.1097/MD.0000000000027519

**Published:** 2021-10-08

**Authors:** Hyungsuk Kim, Koh-Woon Kim, Won-Seok Chung

**Affiliations:** a Department of Clinical Korean Medicine Graduate School, Kyung Hee University, 23 Kyungheedae-ro, Dongdaemun-gu, Seoul, Korea; b Department of Korean Medicine Rehabilitation, Kyung Hee University Medical Center, 23 Kyungheedae-ro, Dongdaemun-gu, Seoul, Korea.

**Keywords:** enhanced recovery after surgery, lumbar disc herniation, protocol, systematic review, traditional Chinese medicine

## Abstract

**Background:**

Patients with lumbar disc herniation, who undergo spine surgery, occasionally complain of pain and functional disability. Fortunately, the concept of enhanced recovery after surgery has emerged recently. As a result, patients seek traditional Chinese medicine after spine surgery. This systematic review will thoroughly analyze and synthesize evidence on integrative traditional Chinese medicine therapy for lumbar disc herniation after surgery.

**Methods:**

The following databases will be utilized to search for pertinent studies: the Cochrane Central Register of Controlled Trials, MEDLINE/PubMed, EMBASE, Chinese National Knowledge Infrastructure, Japan Medical Abstracts Society, and 7 Korean databases (the Korean Studies Information Service System, Korean Association of Medical Journal Editors, National Digital Science Library, Database Periodical Information Academic Korean Traditional Knowledge Portal, Oriental Medicine Advanced Searching Integrated System, and Korean National Assembly Digital Library). The risk of bias of the selected studies will be assessed according to the Cochrane assessment tool for risk of bias. For articles that used the same measurements, a meta-analysis will be conducted to synthesize the results of each trial. Pain severity will be the primary outcome, while the results of functional questionnaires and range of motion, etc, will be the secondary outcomes.

**Results and conclusion:**

Since this protocol does not include any data from patients, ethics approval is not required. The results of this review will be disseminated through a peer-reviewed journal.

**Registration number:**

DOI 10.17605/OSF.IO/KP47A (https://osf.io/kp47a)

## 1 Introduction

People in modern society generally lack exercise and activity and maintain a sedentary life. This has globally resulted in an increase in cases of low back pain. Additionally, the duration of low back pain increased by more than 50% between 1990 and 2015.^[1]^ Low back pain confers disability and lowers the quality of life.^[2]^

Lumbar disc herniation (LDH) is one of the main causes of low back and leg pain. Moreover, it can also manifest as sensory loss or muscle weakness.^[3]^ Around 90% patients have reportedly recovered with conservative treatment.^[4]^ However, the presence of severe pain, regardless of the period of noninvasive care, progressive motor weakness, or cauda equina syndrome, may sometimes require surgical intervention.^[5]^

Surgery for LDH has several advantages over conservative treatment. It provides fast pain relief.^[6]^ Nonetheless, midterm or long-term follow-up do result in better outcomes.^[7]^ Moreover, surgical intervention can sometimes lead to pain or dysfunction of the low back or leg in some patients. At this point, conservative treatment can be helpful for patients not undergoing additional surgery.

Enhanced recovery after surgery (ERAS) is an emerging concept for good postoperative conditions in medical teams and patients.^[8]^ When appropriately applied to patients undergoing spine surgery, ERAS can result in fast pain relief, reduced hospital stay, fast return to everyday life, and low costs.^[9,10]^

Traditional Chinese medicine (TCM) is an effective treatment option for ERAS.^[11]^ In clinical situations, TCM is usually administered in an integrative form of 2 or more TCM interventions, such as acupuncture, moxibustion, cupping, herbal medicine, and tuina.^[12]^ Various interventions can lead to diverse effects. Acupuncture has a positive effect on postoperative pain relief.^[13]^ Moxibustion enhances autophagy and reduces apoptosis, thereby providing beneficial effects to intervertebral disc degeneration.^[14]^ Spinal manipulation affects brain activity in patients with LDH.^[15]^ Therefore, it is hypothesized that a combination of TCM interventions may result in benefits in LDH patients who undergo surgery.

No systematic review has investigated the effect of integrative TCM in patients with LDH after surgery. This systematic review will aim to thoroughly assess and analyze randomized controlled trials (RCTs) on integrative TCM for LDH after surgery.

## 2 Methods

### 2.1 Study registration

This protocol was written according to the preferred reporting items for systematic reviews and meta-analysis protocols,^[16]^ which have been uploaded and registered on the Open Science Framework (osf.io/kp47a).

### 2.2 Eligibility criteria for study selection

#### 2.2.1 Types of studies

RCTs in English, Chinese, Japanese, and Korean languages will be included in this systematic review. Crossover studies, quasi-RCTs, case reports, or laboratory studies will be excluded.

#### 2.2.2 Types of participants

Patients who underwent disc surgery after being diagnosed with LDH will be included. The age, race, and sex will not be considered for discrimination.

#### 2.2.3 Types of interventions

##### 2.2.3.1 Experimental group

Patients who underwent 2 or more TCM interventions, such as acupuncture, moxabustion, cupping, herbal medicine, and tuina will be included.

##### 2.2.3.2 Control group

Patients who underwent conventional medical care will be included in the control group. It includes oral medications, physical therapy, electrical stimulation, and injections, etc.

#### 2.2.4 Types of outcome measures

The primary outcome will be the pain score, which will be quantified by pain scales, such as the visual analogue scale and numerical rating scale. The secondary outcomes will be measured using questionnaires for lumbar function, the quality of life, and the range of motion of the lumbar spine.

### 2.3 Search strategy

#### 2.3.1 Electronic data

The Cochrane Central Register of Controlled Trials, MEDLINE/PubMed, EMBASE, Chinese National Knowledge Infrastructure, Japan Medical Abstracts Society, and 7 Korean databases (the Korean Studies Information Service System, Korean Association of Medical Journal Editors, National Digital Science Library, Database Periodical Information Academic Korean Traditional Knowledge Portal, Oriental Medicine Advanced Searching Integrated System, and Korean National Assembly Digital Library) will be systematically searched for articles from their inception to May 2021. The search strategies are presented in Table 1.

**Table 1:** Search terms for PubMed.

#1	Diskectomy OR spinal fusion OR postlaminectomy OR herniated disc OR herniated disk OR disc prolapse OR disk prolapses OR disc prolapses OR disk prolapse OR intervertebral disc displacement OR intervertebral disc displacements OR intervertebral disk displacement OR intervertebral disk displacements OR slipped disc OR prolapsed disc OR prolapsed disk
#2	Acupuncture OR acupuncture therapy OR pharmacopuncture OR herb acupuncture OR herbal acupuncture OR herbalized acupuncture OR pharmaco-acupuncture OR pharmaco-puncture OR pharmacoacupuncture OR pharmacopuncture OR electroacupuncture OR acupuncture, electric OR electric acupuncture OR electrical acupoint stimulation OR electrical acupuncture OR electro-acupuncture OR electroacupuncture OR electrode acupuncture OR electronic acupuncture OR moxibustion OR moxibustion OR warm acupuncture OR cupping therapy OR cupping (therapy) OR cupping manipulation OR cupping therapy OR cupping treatment OR fire cupping OR flash cupping OR moving cupping OR suction cupping OR vacuum cupping OR Chinese medicine OR Chinese herbal medicine OR Chinese medicine OR medicine, Chinese traditional OR traditional Chinese medicine OR herbal medicine OR botanical medicine OR ethnobotanical medicinal use OR ethnobotanical medicine OR ethnobotanical remedy OR herb medicine OR herbal medicine OR herbal remedy OR medicine, herbal OR phyto-medicine OR phytomedical remedy OR phytomedicine OR plant medicine OR plant-based medicine OR plant-based remedy OR tui na OR tuina
#3	Randomized controlled trials OR randomized controlled trial OR controlled trial, randomized OR randomized controlled study OR randomized controlled trial OR randomized controlled study OR randomized controlled trial OR trial, randomized controlled
#4	#1 AND #2 AND #3‘

#### 2.3.2 Other resources

Reviewers will check the references of the included papers in detail. Manual searches will also be conducted for articles that are not uploaded online.

### 2.4 Data collection and analysis

#### 2.4.1 Study selection

**Figure 1:**
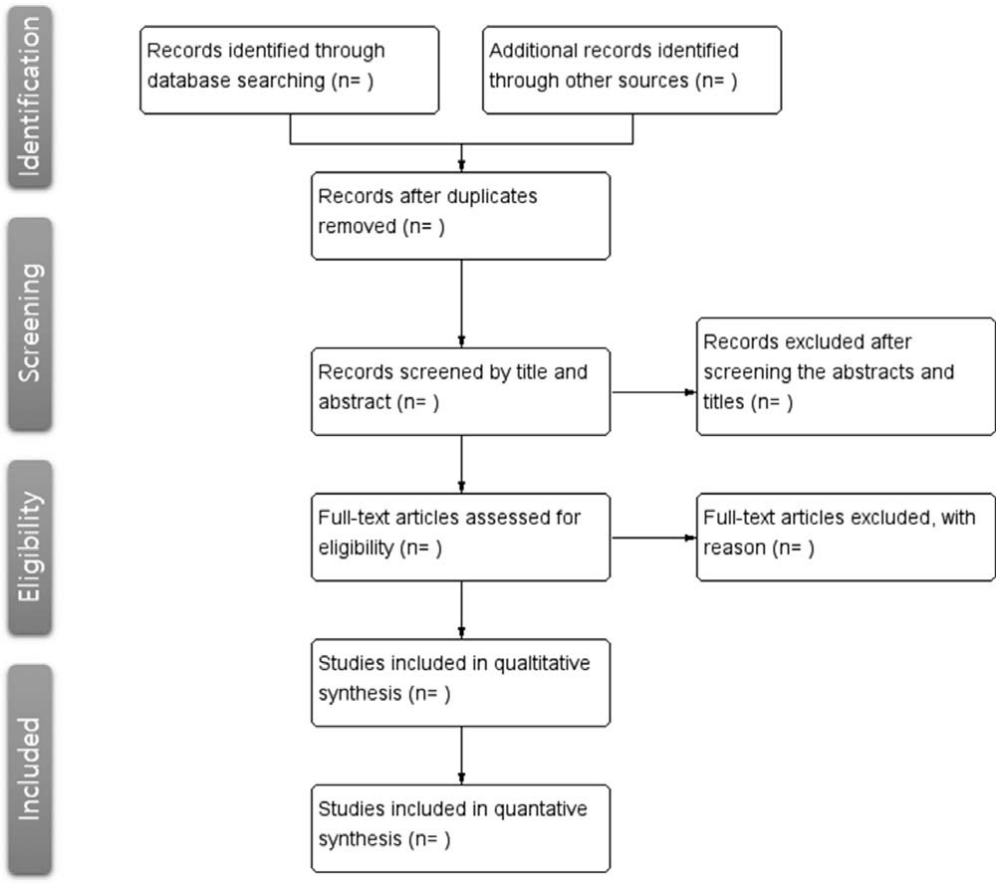
Flowchart of this systematic review.

Two independent researchers (HK and KK) will screen and select the studies. They will repeatedly review the data according to the selection criteria. If they do not agree on a certain issue, another third reviewer (WC) will take part in the decision. A flow diagram based on the preferred reporting items for systematic reviews and meta-analysis flowchart is presented in Figure 1.

#### 2.4.2 Data extraction and management

An Excel file with vacant cells for the title, author name, number of participants, interventions, and outcomes will be provided to the 2 reviewers for data extraction.

#### 2.4.3 Assessment of the risk of bias and quality of included studies

The Cochrane risk of bias assessment tools will be used to assess included studies in seven domains: random sequence generation, allocation concealment, blinding of participants and personnel, blinding of outcome assessment, incomplete outcome data, selective outcome reporting, and other sources of bias. One of the 3 evaluations will be written for each item: “high risk,” “low risk,” or “unclear risk.” Any discrepancies will be resolved by a third reviewer.

#### 2.4.4 Assessment of the effects of treatment

For continuous variables, the mean differences and the 95% confidence intervals will be used.

#### 2.4.5 Management of missing data

If any data are missing or ambiguous information is obtained during the review process, researchers will contact the corresponding author of the article for explanation and clarity.

#### 2.4.6 Data synthesis

When there are articles on the same kinds of TCM interventions, data synthesis will be conducted using the software distributed by the Cochrane Collaboration (The Cochrane Collaboration, Review Manager Software Version 5.3). A random-effects model will be employed when the heterogeneity is high (I^2^ > 50%). Otherwise, a fixed-effects model will be used.

#### 2.4.7 Ethics and dissemination

This is a protocol for a systematic review; therefore, ethical approval is waived. The findings of this systematic review will be disseminated through a peer-reviewed journal for publication.

## 3 Discussion

An increasing number of patients with LDH are treated with surgery.^[17]^ However, several patients still complain of symptoms of low back or leg pain or dysfunction after surgery. Increasing attention has been provided to patients who experience discomfort after surgical intervention since low back pain can result in long hospital stays and high medical costs.^[18]^ These patients seek noninvasive and safe treatment modalities. A systematic review analyzed the performance of acupuncture for postoperative pain in various operations and concluded that acupuncture improved postoperative pain and reduced opioid use.^[19]^ According to a report from a Korean hospital in 2012, 64% of patients who underwent spinal surgery sought TCM as a primary means of treatment.^[20]^ TCM is now widely used clinically. In particular, the combined type of TCM is common. This systematic review will search for RCTs to provide clinicians or patients with an accurate viewpoint of the treatment options for LDH after surgery.

## Author contributions

**Conceptualization:** Hyungsuk Kim, Won Seok Chung.

**Data curation:** Koh-Woon Kim, Won Seok Chung.

**Formal analysis:** Hyungsuk Kim, Koh-Woon Kim.

**Funding acquisition:** Won-Seok Chung.

**Investigation:** Koh-Woon Kim.

**Methodology:** Hyungsuk Kim.

**Project administration:** Hyungsuk Kim, Koh-Woon Kim.

**Resources:** Koh-Woon Kim.

**Software:** Koh-Woon Kim.

**Supervision:** Koh-Woon Kim.

**Validation:** Koh-Woon Kim.

**Visualization:** Koh-Woon Kim, Won Seok Chung.

**Writing – original draft:** Hyungsuk Kim, Won Seok Chung.

**Writing – review & editing:** Hyungsuk Kim, Won Seok Chung.

## References

[1] Hartvigsen J Hancock MJ Kongsted A. What low back pain is and why we need to pay attention. Lancet 2018;391:2356–67. 29573870 10.1016/S0140-6736(18)30480-X

[2] Buchbinder R van Tulder M Oberg B. Low back pain: a call for action. Lancet 2018;391:2384–8. 29573871 10.1016/S0140-6736(18)30488-4

[3] Koes BW van Tulder MW Peul WC. Diagnosis and treatment of sciatica. BMJ 2007;334:1313–7. 17585160 10.1136/bmj.39223.428495.BE PMC1895638

[4] Gibson JN Waddell G. Surgical interventions for lumbar disc prolapse. Cochrane Database Syst Rev 2007;2007:CD001350. 10.1002/14651858.CD001350.pub3 17253457

[5] Gibson JN Waddell G. Surgery for degenerative lumbar spondylosis. Cochrane Database Syst Rev 2005;2005:CD001352. 10.1002/14651858.CD001352.pub2 15846617

[6] Fisher CG Vaccaro AR Patel AA. Evidence-based recommendations for spine surgery. Spine (Phila Pa 1976) 2020;45:851–9. 32355150 10.1097/BRS.0000000000003512

[7] Gugliotta M da Costa BR Dabis E. Surgical versus conservative treatment for lumbar disc herniation: a prospective cohort study. BMJ Open 2016;6:e012938. 10.1136/bmjopen-2016-012938 PMC5223716 28003290

[8] Xin C Sun JH. The value of acupuncture-moxibustion in enhance recovery after surgery. Zhongguo Zhen Jiu 2020;40:679–82. 32538023 10.13703/j.0255-2930.20190501-0005

[9] Elsarrag M Soldozy S Patel P. Enhanced recovery after spine surgery: a systematic review. Neurosurg Focus 2019;46:E3. 10.3171/2019.1.FOCUS18700 30933920

[10] Dietz N Sharma M Adams S. Enhanced recovery after surgery (ERAS) for spine surgery: a systematic review. World Neurosurg 2019;130:415–26. 31276851 10.1016/j.wneu.2019.06.181

[11] Lee J Shin JS Lee YJ. Long-term course of failed back surgery syndrome (FBSS) patients receiving integrative korean medicine treatment: a 1 year prospective observational multicenter study. PLoS One 2017;12:e0170972. 28129399 10.1371/journal.pone.0170972 PMC5271391

[12] Lim B. Korean medicine coverage in the National Health Insurance in Korea: present situation and critical issues. Integr Med Res 2013;2:81–8. 28664058 10.1016/j.imr.2013.06.004 PMC5481697

[13] Acar HV. Acupuncture and related techniques during perioperative period: a literature review. Complement Ther Med 2016;29:48–55. 27912957 10.1016/j.ctim.2016.09.013

[14] Zhang B Zhao Q Li Y Zhang J. Moxibustion alleviates intervertebral disc degeneration via activation of the HIF-1alpha/VEGF pathway in a rat model. Am J Transl Res 2019;11:6221–31. 31632589 PMC6789265

[15] Yuan WA Shen ZB Xue L. Effect of spinal manipulation on brain functional activity in patients with lumbar disc herniation. Zhejiang Da Xue Xue Bao Yi Xue Ban 2015;44:124–30. 26038129 10.3785/j.issn.1008-9292.2015.03.002 PMC10396920

[16] Shamseer L Moher D Clarke M. Preferred reporting items for systematic review and meta-analysis protocols (PRISMA-P) 2015: elaboration and explanation. BMJ 2015;350:g7647. 25555855 10.1136/bmj.g7647

[17] Weinstein JN Lurie JD Olson PR Bronner KK Fisher ES. United States’ trends and regional variations in lumbar spine surgery: 1992–2003. Spine (Phila Pa 1976) 2006;31:2707–14. 17077740 10.1097/01.brs.0000248132.15231.fe PMC2913862

[18] Sebaaly A Lahoud MJ Rizkallah M Kreichati G Kharrat K. Etiology, evaluation, and treatment of failed back surgery syndrome. Asian Spine J 2018;12:574–85. 29879788 10.4184/asj.2018.12.3.574 PMC6002183

[19] Wu MS Chen KH Chen IF. The efficacy of acupuncture in post-operative pain management: a systematic review and meta-analysis. PLoS One 2016;11:e0150367. 26959661 10.1371/journal.pone.0150367 PMC4784927

[20] Choi HS Chi EH Kim MR. Demographic characteristics and medical service use of failed back surgery syndrome patients at an integrated treatment hospital focusing on complementary and alternative medicine: a retrospective review of electronic medical records. Evid Based Complement Alternat Med 2014;2014:714389. 25530787 10.1155/2014/714389 PMC4235193

